# Systematic review of dementia prevalence 1994 to 2000

**DOI:** 10.1590/S1980-57642008DN10300003

**Published:** 2007

**Authors:** Marcos Antonio Lopes, Sérgio Ricardo Hototian, Geraldo C. Reis, Hélio Elkis, Cassio Machado de Campos Bottino

**Affiliations:** 1 Clinics Hospital, Department of Neurology, Psychiatry and Medical Psychology of Faculty of Medicine of Ribeirão Preto, University of São Paulo, Brazil.; 2 Old Age Research Group (PROTER), Institute of Psychiatry, Faculty of Medicine, University of São Paulo, Brazil.; 3 Department of Psychiatry, Faculty of Medicine, University of São Paulo, Brazil.

**Keywords:** epidemiology, prevalence, dementia, Alzheimer disease, review

## Abstract

Ageing has occurred in all regions of the world, with impact on neuropsychiatric disorders, particularly dementia. However, previous meta-analysis and reviews have shown high variability in world dementia prevalence rates.

**Objective:**

The aim of this study was to perform a wide-ranging review of the dementia prevalence studies published in recent years.

**Methods:**

The search was made on Medline, Lilacs and Embase databases for research conducted between 1994 and 2000. The main inclusion criteria were: use of standard diagnostic criteria and investigation of community samples.

**Results:**

The final selection included 42 papers, from all continents. The mean prevalence rate of dementia in subjects aged 65 years and older, for continents, ranged from 2.2% in Africa to 8.9% in Europe, and among countries, from 1.3% in India to 14.9% in Spain. However, there was a trend of clustering of the world prevalence rates with the majority of studies reporting rates between 4.2% and 7.2% (≥65 years). Age directly influenced the rates, with a mean prevalence rate of 1.2% (95% CI: 0.8.1.5) for the 65-69 years group and 39.9% (95% CI: 34.4.45.3) for the 90-94 year group, but showing less pronounced influence in the very elderly age group. The urban samples had higher rates, where no significant gender difference was evidenced.

**Conclusion:**

The age influence over dementia rates apparently leveled off in the very elderly group while a trend toward similar dementia prevalence rates around the world was probably influenced by greater homogeneity in diagnostic criteria.

## Abstract (pt)

população idosa vem aumentando em todo o mundo, levando a um impacto nos transtornos psiquiátricos, principalmente os quadros de demência. Contudo, revisões e meta-análises prévias têm mostrado alta variabilidade nas taxas mundiais de prevalência de demência.

**Objetivo:**

O objetivo deste estudo foi realizar uma ampla revisão de trabalhos recentes de prevalência de demência.

**Métodos:**

O levantamento foi feito em três bases de dados, Medline, Lilacs e EMBASE, no período de 1994 a 2000. Os principais critérios de inclusão foram: uso de critérios diagnósticos padronizados e investigação de amostra comunitária.

**Resultados:**

A seleção final incluiu 42 artigos, de todos os continentes. A taxa média de prevalência de demência, na idade maior ou igual a 65 anos, entre continentes, variou de 2.2% na África, até 8.9% na Europa, e entre países, de 1.3% na Índia, até 14.9% na Espanha. Contudo, a maioria dos estudos reportou taxas entre 4.2% e 7.2% (≥65 anos). A idade exerceu influência direta sobre as taxas de prevalência, com taxas médias de 1.2% na faixa etária 65-69 anos, e 39.9% na faixa etária 90-94 anos, mostrando, no entanto, um efeito menos pronunciado nos idosos muito idosos. A população de procedência urbana teve taxas significativamente maiores, e não houve diferença significativa entre os sexos.

**Conclusão:**

A influência da idade sobre as taxas de prevalência de demência diminuiu entre os idosos mais “idosos”, e a tendência de resultados similares em todo o mundo provavelmente foi influenciada pela homogeneidade nos critérios diagnósticos.

The world ageing process impacts certain diseases, and in recent years researchers from many countries have studied the prevalence and risk factors associated with dementia, especially Alzheimer disease (AD).

A significant number of dementia prevalence studies have been carried out in recent decades and published data have been analyzed by reviews and meta-analyses. Since the influential review published by Jorm et al. (1987),^1^ which showed the strong effect of age on dementia prevalence, the descriptive review of Fratiglioni et al. (1999),^2^ reporting that Vascular Dementia (VD) is more prevalent than Alzheimer disease in Asian countries and the European review by Lobo et al. (2000),^3^ observing Alzheimer disease as the major cause for the fast increase of dementia in the elderly, a high variability of dementia prevalence rates has been observed around the world. However previous reviews have either shown studies with broad diagnoses or specific characteristics (such as same region) or have evaluated a single variable. A wide-ranging review which evaluates recent studies and has high homogeneity in research methods, including diagnostic criteria, can help researchers to understand the factors influencing the distribution of dementia rates throughout different regions of the world.

The purposes of this study were:

1) to review worldwide dementia prevalence studies from 1994 to 2000;
2) to assess and to integrate dementia prevalence rates, evaluating its distribution related to demographic factors;
3) to assess prevalence rates of both main etiological subtypes of Dementia, namely: Alzheimer disease and Vascular Dementia;
4) to assess the influence of methodological variables on dementia prevalence rates;
5) to analyse the relationship between dementia prevalence and the variables studied, in order to detect possible associations which could be addressed in future researches.

A preliminary analysis of the data presented in this article, without statistical analysis, was published previously.^4^

## Methods

### Survey of studies

Studies published between 1994 and 2000 were obtained from 3 data bases: Medline, Lilacs and EMBASE. In step one all languages were considered. The terms “epidemiology”, “prevalence” and “dementia” were used in “all fields”. All articles were sent for selection, according to specific criteria. Two examiners working independently performed the selection on the Medline database (M.A.L. and S.R.H).

### Selection of studies

Studies were selected in 3 phases, with the following inclusion criteria:

- **1^st^ phase:** Original articles that have estimated dementia prevalence in community samples using internationally accepted diagnostic criteria for “Dementia Syndrome”, according to most recent diagnostic manuals, chiefly the DSM-III-R, DSM-IV, ICD-10 or similar (very broad diagnoses were excluded, such as “organic cerebral syndrome” or “organic mental disorder”).
- **2^nd^ Phase:** Articles written in English, Portuguese, and Spanish; accessibility.
- **3^rd^ Phase:** Data presented in a way that permitted comparison between studies.

### Characterization of the studies evaluated

The following variables were recorded for each study: authors, country of origin, publication year, age range studied, sample composition (inclusion of institutionalized subjects, urban or rural provenance), total sample size, loss of subjects, sub-sample size by age group and gender, dementia prevalence in elderly aged 65 years and older and at 5-year intervals, prevalence in each gender and age interval, prevalence of Alzheimer disease and Vascular Dementia etiological diagnoses, diagnostic criteria, use of computerized tomography and laboratorial exams.

### Data investigation

#### Descriptive analysis

The studies were initially gathered on six geographic areas. Crude mean prevalence and distribution with regard to age group, gender and etiology were evaluated for each geographic area, within each country and among the countries. Although the means were not adjusted, only studies with prevalence rates in elderly aged 65 years and older were taken into account. Exception was made when analysing the age variable, whereby only those studies with specific age ranges (65-69, 70-74 and so on) were investigated, and for the etiology variable for which 3 studies with age range of 100 years and older were separately analysed.

#### Statistical analysis

Univariate analysis: initially, the age effect over prevalence rates was examined, and three statistics models that could best represent the data were tested, following the methodology used by Ritchie and Kildea (1995).^5^ Three functions were applied – exponential, logistic and modified logistic – to obtain a general model that would be able to estimate the relationship between the variables “dementia prevalence” and “age”. The mean prevalence for each age group was calculated for this procedure and adjustments were made for the size of each age range (number of subjects). Age information was based on age group intervals defined in the studies (65-69, 70-74, 75-79...), and were codified in the present analysis from the approximate midpoint (67.5, 72.5, 77.5...). R^2^ was calculated for each function (this statistics gives a percent variation of the prevalence “explained” by age, and can be used both for verifying if the adjustment is adequate or not, and for comparison between different adjustments). Following the univariable analysis, the mean of each category of a variable was calculated and compared with the mean of other categories of the same variable. According to this strategy, the analysis of the variables “sample type” (community or institution+community), “provenance” (urban or rural), and “gender” was performed. Additionally, an adjustment to the variable “age” was made, where each variable cited above was investigated for each age group intervals. The Bonferroni method was then applied, in which the 0.05 p value was divided by 21 (3 variables and 7 age ranges) in order to obtain a new p value corresponding to the statistical significance. Without the adjustment for the “age” variable, the variables “sample size”, “percentage of participation” and “percentage of loss” were also examined. Non-parametric tests were used, considering that the data did not show a normal distribution. The Mann-Whitney test was used for the variables with two categories such as “sample type” (community, community+institution), and “gender”. The Kruskal-Wallis test was used for the variable with three categories namely, “provenance” (urban, urban+rural, rural) while the Spearman non-parametric correlation coefficient was applied for continuous variables such as “sample size”, “percentage of participation” and “percentage of loss”. The same tests outlined above were used in the analysis of the variables “sample type, “gender” and “provenance’, for all age group intervals.

#### Multivariate analysis

Finally, multivariable analysis was performed using the variables “age’, “gender”, “sample type”, “provenance”, “use of computerized tomography”, “use of laboratorial exams”, “use of total population”, “use of DSM”,^6-8^ “use of Hachinski Ischemic Scale”9 and “use of NINCDS-ADRDA diagnostics criteria”.^10^ The variable “use of NINCDS-ADRDA criteria” was chosen according to the methodology described in the reviews, as the possible interference of diagnostic criteria in dementia prevalence rates was considered. Logistic regression was performed with the Proc Logistic section of the SAS program. This choice of mathematical model took into consideration that the dependent variable was dichotomic – “with dementia” or “without dementia” – and that many independent variables could have potential influence on dementia prevalence rate, as described above. The data were considered for Logistic Regression following this strategy: lines for all age ranges of all studies; each two lines referred to the same age range and the same study, one line for male and another line for female; the first column for the “age” variable (age range); second column for number of cases of dementia; third column for total number of people; the next columns for the other variables. Adjusted “odds ratios” were estimated, with the respective confidence intervals and ‘p’ values.

## Results

### Selection and utilization of studies

1750 articles were found, from which 73 were initially selected, according to the criteria established in the first phase of the selection. Eight articles in other languages were excluded (Dutch: four; Japanese: two; German: one; and Polish: one), and two articles could not be accessed (2^nd^ Phase). In the third phase of the selection, 15 articles were excluded: 12, for using age group different from “65 years and older”, or age intervals different from the majority of other studies; two, for examining specific samples (male gender, retired); and one, for estimating prevalence of moderate and severe degrees of dementia only. In total, 42 articles were finally studied, as shown in Table 1.

**Table 1:** Worldwide dementia prevalence studies, from 1994 to 2000.

Author	Year	Country	Sample	Age	Prevalence (%)	Diagnostic^†^										
Ott et al.^11^	1995	Holland	7528	≥ 55	6.3	a		c								
Prencipe et al.^12^	1996	Italy	1022	≥ 65	8.0	a		c			f		h			
Boersma et al.^13^	1998	Holland	2191	≥ 65	6.5	a			d							
Brayne et al.^14^	1998	England	2622	≥ 65	6.6					e						k
Andersen et al.^15^	1997	Denmark	3346	≥ 65	7.1	a		c			f					
Roelands et al.^16^	1994	Belgium	1736	≥ 65	9.0	a			d							
Lobo et al.^17^	1995	Spain	1134	≥ 65	5.5	a				e						
Pousa et al.^18^	1995	Spain	273	≥ 65	13.9				d							
Pi et al.^19^	1996	Spain	516	≥ 65	14.9	a						g				
Manubens et al.^20^	1995	Spain	1127	≥ 70	17.7	a		c	d	e						
Wernicke and Reischies^21^	1994	Germany	156	≥ 70	11.7	a										
DAlessandro et al.^22^	1996	Italy	365	≥ 75	21.9	a				e	f					
Azzimondi et al.^23^	1998	Italy	773	≥ 75	28.4	a				e	f					
Strauss et al.^24^	1999	Sweden	1848	≥ 77	19.3^‡^	a				e	f					
Fichter et al.^25^	1995	Germany	402	≥ 85	27.8	a	b									
Ravaglia et al.^26^	1999	Italy	154	≥ 100	61.9	a	b	c			f					
Shaji et al.^27^	1996	India	2067	≥ 60	3.1	a	b									
Yamada et al.^28^	1999	Japan	2934	≥ 60	7.2	a		c		e	f					
Komahashi et al.^29^	1994	Japan	2778	≥ 65	6.1	a				e						
Kiyohara et al.^30^	1994	Japan	1189	≥ 65	5.5^‡^	a										
Ogura et al.^31^	1995	Japan	3524	≥ 65	6.7	a		c		e						
Shiba et al.^32^	1999	Japan	201	≥ 65	8.5	a		c		e						
Chandra et al.^33^	1998	India	536	≥ 65	1.3						f			i		
Park et al.^34^	1994	Korea	766	≥ 65	10.8	a				e						
Woo et al.^35^	1998	Korea	2171	≥ 65	9.5	a		c		e						
Liu et al.^36^	1996	Taiwan	1200	≥ 65	4.4	a		c					h			
Lin et al.^37^	1998	Taiwan	4015	≥ 65	3.7	b		c		e			h	i		
Liu et al.^38^	1995	Taiwan	5297	≥ 65	2.0	a										
Chiu et al.^39^	1998	Hong Kong	1034	≥ 70	6.1	a			d							
Asada et al.^40^	1996	Japan	47	≥ 100	70.2	a	b	c								
Hendrie et al.^41^	1995	USA	4812	≥ 65	4.8	a	b	c								
Beard et al.^42^	1995	USA	^§^REP	≥ 65	5.7										j	
Graves et al.^43^	1996	USA	1985	≥ 65	6.3	a		c			f					
Fillenbaum et al.^44^	1998	USA	4236	≥ 65	7.0-7.2	a		c								
Breitner et al.^45^	1999	USA	5677	≥ 65	9.6	a		c					h	i		
McDowell et al.^46^	1994	Canadá	10263	≥ 65	4.2	a	b	c								
Ebly et al.^47^	1994	Canadá	4510	≥ 85	28.5	a	b	c		e						
Powell^48^	1994	USA	40	≥ 100	57.5						f					
Farrag et al.^49^	1998	Egypt	2000	≥ 60	4.5	a		c		e						
Ogunniyi et al.^50^	1997	Nigeria	2494	≥ 65	2.2	a	b	c								
Herrera Jr. et al.^51^	1998	Brazil	1660	≥ 65	7.1	a		c		e	f		h			
Henderson et al.^52^	1994	Australia	1377	≥ 70	7.3^‡^	a	b									

† Diagnostic Criteria and Instruments: (a) "Diagnostic and statistical manual of mental disorders, 3rd ed. rev" (DSM-III-R)^7^ ou "Diagnostic and statistical manual of mental disorders, 4^th^ ed." (DSM-IV);^8^ (b) International of Classification of Diseases ICD-1053; (c) "National Institute of Neurological and Communicative Disorders and Stroke and the Alzheimer's Disease and Related Disorders Association (NINCDS-ADRDA)";^10^ (d) "Cambridge Mental Disorders of the Elderly Examination" (CAMDEX);^54^ (e) Hachinski Ischemic Scale (HIS);^9^ (f) "Clinical Dementia Rating" (CDR);^55^ (g) "Blessed Dementia Scale" (BDS);^56^ (h) "National Institute of Neurological Disorders and Stroke and Association Internationale pour la Recherche et l'Enseignement en Neurosciences (NINDS-AIREN);^57^ (i) "The Consortium to Establish a Registry for Alzheimer's Disease" (CERAD);^58^ (j) "Rochester Epidemiologic Project" (REP); (k) AGECAT (Automated Geriatric Examination for Computer Assisted Taxonomy).^59^

‡ Crude rate.

§ Linear interpolation from decennial census data (1970 to 1990).

The studies were partly used in that, for each evaluation, only one set of articles was used since not all the data studied in these articles were available. In the descriptive analysis for variables “prevalence of dementia”, “age”, “gender” and “Alzheimer disease / Vascular Dementia Ratio”, 26^12-19,29-38,41-46,50,51^, 26^11-18,20-24,25,27,29,31,32,34,37,39,43,45,47,51,52^, 19^13-15,17-19,29-32,34-38,43,45,46,51^ and 2115-^18,26,29-32,34-38,40,41,43,46,48,50,51^ articles out of the total number of articles selected were used, respectively.

### Descriptive analysis

The distribution by geographic area was defined as follows:

- **Europe:** 16 studies (Spain: four, Italy: four, Holland: two, Germany: two, England: one, Sweden: one, Denmark: one, Belgium: one).
- **Asia:** 14 studies (Japan: six, Taiwan: three, Korea: two, India: two, Hong-Kong: one).
- **North America:** 8 studies (United States: six, Canada: two).
- **Africa:** 2 studies (Nigeria: one, Egypt: one).
- **South America:** 1 study (Brazil: one).
- **Oceania:** 1 study (Australia: one).

### Social-demographic distribution

The mean dementia prevalence rates, in elderly aged 65 years and older, were 2.2% in Africa, 5.8% in Asia, 6.2% in North America, 7.1% in South America, up to 8.9% in Europe. In Nigeria, one of the lowest results was described, 2.2%^50^, below rates in the American study^41^ that included subjects classified as “African-American”.

Studies performed in Asian countries presented great variability in their results, in the same country and among different countries. For example, in Taiwan the mean prevalence was 3.3%, with results varying between 2.0%^38^ and 4.4%.^36^ In Japan, the mean prevalence was 6.7%, with results ranging from 5.5%30 to 8.5%,^32^ and in Korea the mean prevalence was 10.1%, with the lowest variation among the studies reviewed. The highest result among Japanese studies (8.5%)^32^ occurred in a rural region. The highest prevalence in Asian countries was 10.8%,^34^ also in a rural zone, in a very economically limited Korean community.

Great variability was found in the North American results. The prevalence varied between 4.8%^41^ and 9.6%,^45^ with a mean of 6.7%. Among investigations, Fillenbaum et al. (1998)^44^ and Hendrie et al. (1995)^41^ studies are noteworthy.

In the first study, almost no differences were observed between African and White Americans (7.0 and 7.2% respectively), while in the second study, a high prevalence in African-American subjects was described (4.8%) compared with rates for Africans living in Africa (2.2%).^41,50^ The Canadian study described a prevalence of 4.2%.^46^

The results of European studies, estimating prevalence in 65 years and older, were as follows: Holland: 6.5%;^13^ England: 6.6%;^14^ Denmark: 7.1%;^15^ Italy: 8.0%;^12^ Belgium: 9.0%16 and Spain: 5.5%,^17^ 13.9%18 and 14.9%.^19^ The mean in Europe was very high at 8.9%, strongly influenced by two Spanish studies,^18,19^ that investigated rural and rural/ urban population, and found the highest rates of all the European studies.

In the Brazilian study, the only study selected in South America, the authors described a rate of dementia prevalence of 7.1%.^51^

**Figure 1:**
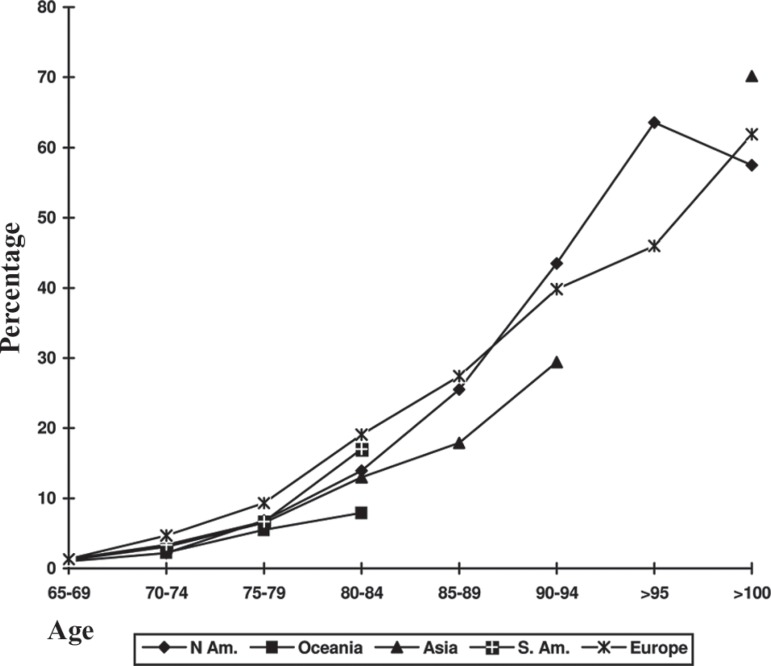
Dementia prevalence rate (mean), in each region.

Dementia prevalence increased with age in all regions, except North America (a study conducted in the USA)^48^, for the group aged 100 years and older, where a lower rate was noted compared to the preceding age group (Figure 1). Mean rates of dementia prevalence, worldwide and by region, did not increase at such a high rate in the oldest age groups compared to other age groups (Table 2).

**Table 2:** Effect of age over dementia prevalence rates. Crude mean rate in each age group (%) and amount of the increase of rates, as age increases (times).

	N	Mean		Increase
Age	(studies)	rate (%)^†^	(95% CI)	of rates^‡^
65-69	17	1.2	(0.8-1.5)	
70-74	19	3.7	(2.6-4.7)	3.0
75-79	21	7.9	(6.2-9.5)	2.1
80-84	20	16.4	(13.8-18.9)	2.0
85-89	16	24.6	(20.5-28.6)	1.5
90-94	6	39.9	(34.4-45.3)	1.6
>95	6	54.8	(45.6-63.9)	1.3

† Mean dementia prevalence rate;

‡ Amount of increase of rates, in comparison with rate before.

Higher dementia prevalence was observed in the female gender, across all regions studied. In some studies the ratio was two women to every one man. However, approximately 25% of the studies described higher prevalence in male gender with these differences not reaching statistical significance. Fillenbaum et al. (1998)^44^ observed a slight predominance of the male gender, which was not statistically significant, in dementia cases for African-Americans, in contrast to the result observed for White Americans. Ogunniyi et al. (1997),^50^ in Nigeria, found an overwhelming prevalence of male gender (6:2 ratio) in Vascular Dementia cases.

**Figure 2:**
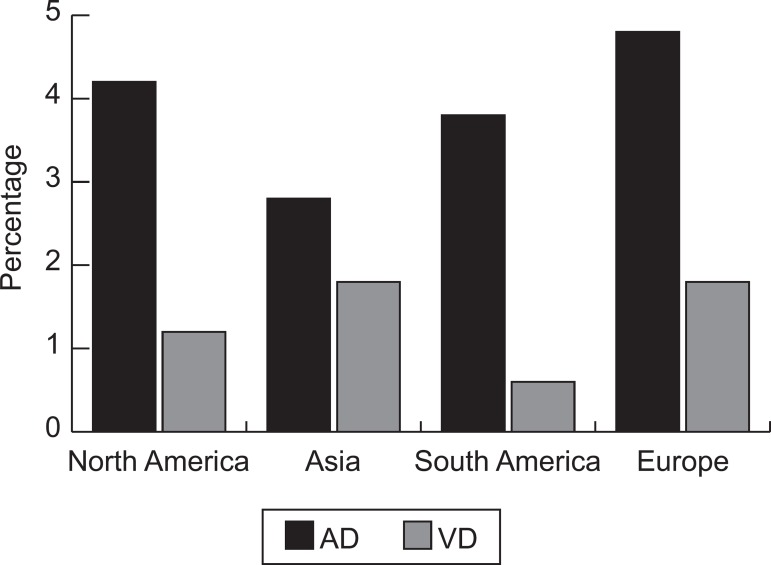
Crude mean rate of Alzheimer’s disease (AD) and vascular dementia (VD) in each region, in elderly aged 65 years and older.

**Figure 3:**
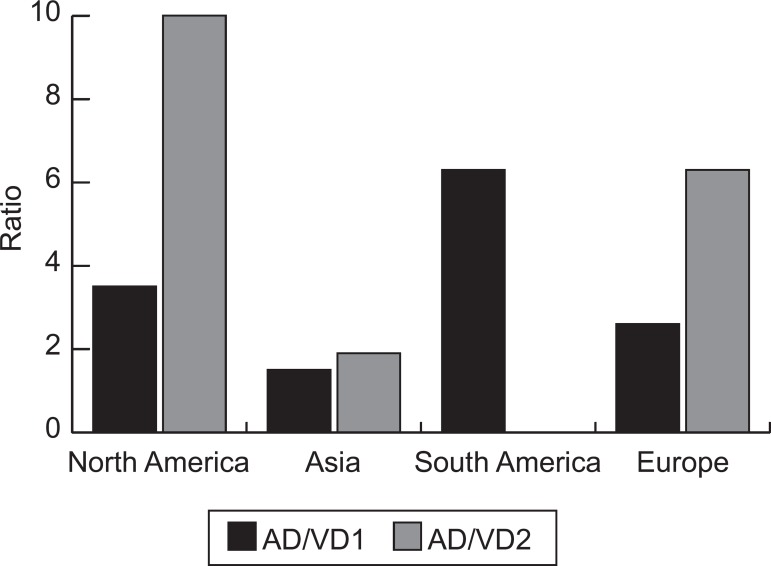
Ratio Alzheimer’s disease (AD) and vascular dementia (VD) in each region, in elderly aged 65 years and older (AD/VD 1) and in 100 years and older (AD/VD 2).

Prevalence of Alzheimer disease was higher than Vascular Dementia in all researched regions, particularly in the South-American study^51^ which presented results two to three times higher than in other regions. An important inversion of this relationship occurred in 25 % of the Asian studies, with VD prevalence described in two studies as two times greater than AD. The age effect over these two etiologic diagnoses was also evident, verifying that the AD/VD ratio showed an important increase in centenarians (Figures 2 and 3).

Predominance of Vascular Dementia over Alzheimer disease, identified as a tendency in Japan by Komahashi et al. (1994),^29^ was not observed in the Ogura et al. (1995) study,^31^ where an Alzheimer disease/Vascular Dementia ratio of 1.5:1 was found. Kiyohara et al. (1994),^30^ despite reporting a higher prevalence of VD over AD, verified a significant increase in AD/VD rate over a 7-year-period.

The AD/VD ratio among African-Americans was similar to those in most of the Western world studies, standing at around 1.5:1. When African-Americans were compared to White Americans, the results were also equivalent.

Approximately 30% of the studies used solely the Mini- Mental State Examination” (MMSE) (Folstein et al., 1975),^60^ for detection of probable dementia cases. Eighty-four percent of the studies utilized criteria from the “Diagnostic and Statistical Manual of Mental Disorders”, from the American Psychiatry Association (versions III-R or IV),^7,8^ and 50% of these studies also used the NINCDS-ADRDA criteria.^10^ Fifty-four percent of the studies used at least one scale or additional criteria for Vascular Dementia (Hachinski Ischemic Scale,^9^ in the majority of the studies, or NINDS-AIREN^57^).

### Statistical analysis

#### Univariate analysis

**Figure 4:**
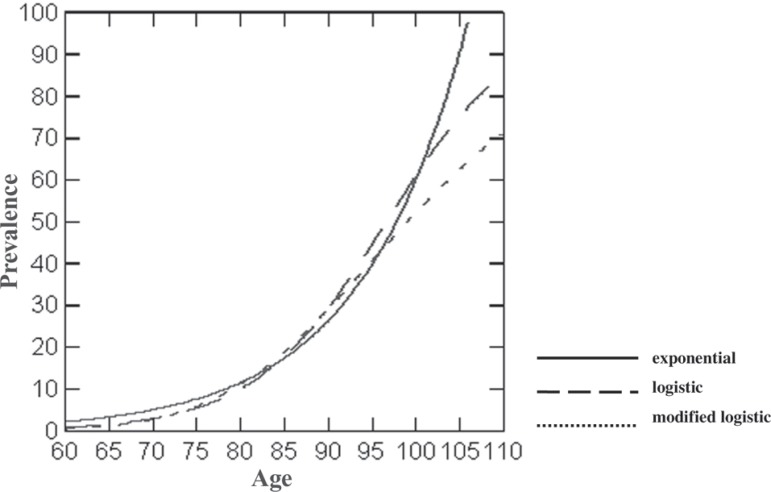
Age influence over dementia prevalence exponential, logistig and modified logistic models.

*Age* – The estimated average prevalence rate and the examined functions, for each age group interval, can be seen in Table 3. All three models permitted excellent adjustments, both by R^2^ values and by estimated values, but the modified logistic was the most efficient, especially when estimated values for each model were compared. It was also noted that the modified logistic model yielded prevalence estimates closer to observed rates. In contrast to the exponential model, both logistic and the modified logistic models presented an inflection point on the resulting graphic (Figure 4), the inflection point in the modified logistic model being prior to the one in the logistic model. In the modified logistic model the highest prevalence rates were reached for higher ages than in the logistic model.

**Table 3:** Effect of age over dementia prevalence rates, according to three statistical models. Rates in observed prevalence and in three models are expressed in percentage (%).

Age^†^ (years)	Prevalence (%)	Exponential	Logistic	Modified logistic
67.5	1.75	4.01	2.04	2.01
72.5	3.84	6.10	3.81	4.42
77.5	7.55	9.18	6.98	8.79
82.5	14.74	13.81	12.46	15.36
87.5	24.08	20.78	21.25	26.43
95.5	37.98	39.95	42.90	41.01
R^2‡^ (%)		96.7	98.2	99.2

† Midpoint between 65-69, 70-74, 75-79, 80-84, 85-89, 90-100;

‡ Refers to amount changes as function of age.

*Sample type (community or institution), provenance (urban or rural), gender* – Studies investigating community+ institution samples and samples from urban provenance, presented significantly higher prevalence rates compared to community alone and samples from urban-rural provenance, respectively. The female gender presented higher rates than male, but this was not statistically significant (Table 4). There was one negative correlation between prevalence rates and the variables “sample size”. (r: –0.62; p<0.01; statistically significant) and “participation rate” (r: –0.41; p:0.06; tendency of association).

**Table 4:** Effect of 'sample' (community or community + institution), 'provenace' (urban or urban+rural or rural) and 'gender' over rates.

Variable	Mean (%)	Median (%)	SD	Statistic p
Community	10.8	8.0	10.5	Mann-Whitney
Com.+Instit.	17.3	12.2	16.6	0.03^†^
Urban	18.1	11.3	17.3	Kruskal-Wallis
Urban+rural	8.5	5.4	8.7	0.02^†^
Rural	12.8	10.0	11.9	
Female	15.8	10.7	16.3	Mann-Whitney
Male	12.4	9.1	11.9	0.48

† Significant statistically.

When the variables “sample type”, “provenance” and “gender” were analysed in each age group, no statistically significant association was found.

#### Multivariate analysis

The logistic regression method allowed analysis of how “age’, “gender”, “sample type”, “provenance”, “use of computerized tomography”, “use of laboratorial exams”, “use of total population”, “use of DSM”,^6-8^ “use of Hachinski Ischemic Scale”^9^ and “use of NINCDS-ADRDA diagnostic criteria”^10^ influenced prevalence rates, and adjusted for each other. Age influenced prevalence rates significantly where age and prevalence rates increased hand-in-hand. Studies with community+institution sample type, rural provenance, “use of total population” and “use of laboratorial exams” presented significantly higher prevalence rates. Studies that used computerized tomography and Hachinski Ischemic Scale presented significantly lower prevalence rates. Variables such as “gender”, “use of DSM” and “use of NINCDS-ADRDA”, did not present statistically significant differences (Table 5).

**Table 5:** Effect of demographic and clinical variables over rates, after multivariate analysis.

Variable	Odds ratio adjusted	C.L. 95%	p
Age	1.15	1.14-1.16	<0.0001^†^
Female	1.10	0.99-1.22	0.0557
Community + institution	1.31	1.00-1.69	0.0426^†^
Urban	1.76	1.39-2.22	<0.0001^†^
Rural	1.38	1.14-1.68	<0.0001^†^
Computerized tomography	0.50	0.39-0.65	<0.0001^†^
Laboratory	1.51	1.13-2.02	0.0053^†^
All population	1.28	1.07-1.53	0.0071^†^
DSM	0.99	0.75-1.30	0.9494
Hachinski ischemic scale	0.71	0.59-0.87	0.0009^†^
NINCDS-ADRDA	1.08	0.92-1.28	0.3143

† Significant statistically.

## Discussion

Methodological advances were evident from the great number of studies that used standardized diagnostic criteria for diagnoses of dementia and its main causes: Alzheimer disease and Vascular Dementia. On the other hand, with relation to the method for detection of probable cases of dementia, the “Mini-Mental of State Examination” (MMSE)^60^ was applied alone in a considerable number of studies. Such strategy is questionable, in view of the effects of age and schooling on the performance of the MMSE.

Results differed significantly both within the same region, and within the same country. Greatest differences within the same region appeared in Asia, where Chandra et al. (1998)^33^ described a prevalence rate of 1.3% in India, while Park et al. (1994)^34^ reported a rate of 10.8% in Korea. However, 14 of the 26 studies reporting dementia prevalence in subjects aged 65 years and older, investigating a total of 40.940 elderly in the community, reported rates ranging from 4.2% to 7.2%. Similarly, there seemed to be a worldwide tendency to report similar rates of dementia prevalence in elderly aged 65 and older (the 14 above-mentioned studies were performed in all researched regions, except Africa). Another illustration of worldwide similarity was the increased rates of prevalence of dementia correlated with increased age in all researched regions (except Africa, due to insufficient data for comparison). There was insufficient evidence to elucidate the causes underlying the extreme dementia rates mentioned, or to assure the available data indicated “true” regional differences, or differences caused by methodological bias.

The results on the variable provenance were controversial. While in the descriptive analysis higher prevalence rates among Japanese,^32^ Asian^34^ and European^19^ subjects were found in rural samples, on the univariable and multivariable analyses, higher prevalence rates were associated to urban provenance as was stated by Jorm et al. (1987).^1^ Regarding the descriptive analysis, it is important to point out the small sample size in the two Spanish studies^18,19^ (with the highest rates found in Europe), which increased the risk of inaccuracy of the results obtained. Controlling the interference of the variables reported in the present review, other factors could possibly explain the association between urban population and higher dementia rates such as lower mortality and other conditions that might be typical of rural populations, but which can also be present in elderly samples living in urban areas, such as poor education^12,22,23,34,35^ (intrinsic factor and not a methodological bias), inadequate mental stimulation,^34^ manual occupation in the past^23^ (closely related to the previous factor), poor diet,^34^ limited access to medical care.^32^

In the present study, the descriptive analysis of the gender variable confirmed a tendency described earlier by Jorm et al. (1987)^1^ and recently by Lobo et al. (2000).^3^ The higher prevalence of dementia in the female gender resulted mainly from the distribution of Alzheimer disease in 75% of the studies reviewed. However, the multivariable analysis did not demonstrate a significant difference between the variable “gender” and prevalence rates, although the “p” value was close to 0.05, indicating a tendency of higher prevalence rates for the female gender. It is not possible to explain whether there is a higher risk for women of developing Alzheimer disease by exclusively examining prevalence studies. Hebert et al. (2001),^61^ in an interesting study compared prevalence and incidence of Alzheimer disease between two populations resident in Boston, USA. The results demonstrated that incidence and prevalence of Alzheimer disease, controlling for age, were not significantly different between men and women. In conclusion, the authors declared that higher prevalence rates of women with Alzheimer disease could be due to women’s higher life span, and not related to any specific risk factor associated to gender.

The use of diagnostic instruments did not influence uniformly the rates of prevalence of dementia, similar to the findings reported by Corrada et al. (1995),^62^ in the study of sources of variability on rates of prevalence of Alzheimer disease. However, the argument that Corrada et al. (1995)^62^ presented, that computerized tomography and the Hachinski Ischemic Scale^9^ contributed to diagnosing cases of Vascular Dementia in subjects with Alzheimer disease, lowering their prevalence rates, could not be applied to the results of the present study, which investigated the influence on prevalence rates of dementia, and not Alzheimer disease. Thus, it raised the hypothesis that factors other than the relationship between Vascular Dementia and Alzheimer disease could explain that association.

The relationship Alzheimer disease/Vascular Dementia appeared considerably higher in South America.^51^ This finding may have been influenced by two main aspects: a “true” low prevalence of Vascular Dementia in this sample, and the fact that this study was one of the few to diagnose, among etiological types of dementia, mixed dementia (Alzheimer disease associated with Vascular Dementia), which may in turn have lowered the rates of Vascular Dementia. However, additional studies in South America are necessary to investigate the distribution of etiological types of dementia in communities from the region.

Increased Alzheimer disease/Vascular Dementia ratio in centenarians indicates a higher influence of age on cases of Alzheimer disease, in comparison with cases of Vascular Dementia, supporting the findings of Jorm et al. (1987)^1^ and Hebert and Brayne (1995).^63^ Two Japanese studies described results that illustrated a historical change in the Alzheimer disease/Vascular Disease ratio in Japan, as Guk- Hee and Ajit (2001)^64^ had previously stated. The study by Ogura et al. (1995)^31^ indicated an inversion of this ratio, observed in earlier studies that demonstrated predominance of Vascular Dementia, perhaps explained by lower rates of cardiovascular diseases in the population studied. In the study by Kiyohara et al. (1994),^30^ the Alzheimer disease/Vascular Dementia ratio showed an increase over a time frame of seven years, possibly because rates of Cerebrovascular Accidents among men in the studied population had lowered. However, it is necessary to point out that the analysis of dementia subtypes must be considered cautiously given approximately 55% of the viable studies for this purpose used specific instruments (NINCDS-ADRDA, NINDS-AIREN or Hachinski Ischemic Scale) while only 25% performed computerized tomography and laboratory studies.

Age also influenced rates of prevalence across all analyses performed, but not homogeneously. Supporting the results of the descriptive analysis for the age variable, the statistical models tested indicated a decrease in the influence of age in older age groups, as illustrated in the modified logistic model. This result confirmed the meta-analysis published by Ritchie and Kildea (1995)^5^ which stated that the prevalence rates of dementia did not increase exponentially as age increases, as Jorm et al. (1987)^1^ had suggested previously. The data available allowed the supposition that there might be a plateau of prevalence rates above the age of 90 years old. Nevertheless, it is important to bear in mind the hypotheses pointed out by Johanson and Zarit (1995)^65^ – that “mortality could potentially ‘lower’ the rates of prevalence of dementia in older age ranges” - and by McGee and Brayne (1998)^66^ - “once mortality is taken into account, incidence must continue to rise into the oldest groups despite the apparent leveling off of prevalence in the very oldest”. Moreover, a recent article by McGee and Brayne (2001),^67^ using a deterministic model, found that only combining extreme changes in mortality, differential mortality and prevalence could lessen the incidence of dementia with increasing age, corroborating the need for more information on the oldest elderly subgroups.

With regard to the considerations presented, the main constraint of this study is the lack of access to a greater amount of data from the studies reviewed. Greater access, allied to systematic examination of incidence studies, could give access to additional information concerning the circumstances that determined the distribution of cases of dementia in the world population.

Our study presented results similar to those of earlier reviews, especially concerning the increase in rates of dementia prevalence parallel to increased age. Regarding this issue, although older elderly were scarce in such studies, there to the assumption that dementia was not related to aging, but to age, as Ritchie and Kildea (1995)^5^ have previously suggested. However, in order to better investigate the relationship between age and dementia rates, it would be essential to control incidence and mortality rates, as pointed out by McGee and Brayne (1998, 2001).^66,67^

The tendency toward a clustering of prevalence rates may have occurred in majority of the studies investigated due to greater homogeneity in diagnostic criteria employed in the studies published between 1994 and 2000.

However, specific regional aspects do exist, such as racial composition, and socio-cultural heterogeneity, especially in regions that have been poorly studied, such as South America and Africa, which justify additional studies on the prevalence and incidence of dementia in developing countries.
